# Counteracting CAR T cell dysfunction

**DOI:** 10.1038/s41388-020-01501-x

**Published:** 2021-01-14

**Authors:** Mansour Poorebrahim, Jeroen Melief, Yago Pico de Coaña, Stina L. Wickström, Angel Cid-Arregui, Rolf Kiessling

**Affiliations:** 1 Department of Oncology–Pathology, Karolinska Institutet, Stockholm, Sweden; 2 Targeted Tumor Vaccines Group, Clinical Cooperation Unit Applied Tumor Immunity, German Cancer Research Center (DKFZ), Heidelberg, Germany

**Keywords:** Cancer immunotherapy, Immunotherapy

## Abstract

In spite of high rates of complete remission following chimeric antigen receptor (CAR) T cell therapy, the efficacy of this approach is limited by generation of dysfunctional CAR T cells in vivo, conceivably induced by immunosuppressive tumor microenvironment (TME) and excessive antigen exposure. Exhaustion and senescence are two critical dysfunctional states that impose a pivotal hurdle for successful CAR T cell therapies. Recently, modified CAR T cells with an “exhaustion-resistant” phenotype have shown superior antitumor functions and prolonged lifespan. In addition, several studies have indicated the feasibility of senescence delay in CAR T cells. Here, we review the latest reports regarding blockade of CAR T cell exhaustion and senescence with a particular focus on the exhaustion-inducing pathways. Subsequently, we describe what potential these latest insights offer for boosting the potency of adoptive cell transfer (ACT) therapies involving CAR T cells. Furthermore, we discuss how induction of costimulation, cytokine exposure, and TME modulation can impact on CAR T cell efficacy and persistence, while potential safety issues associated with reinvigorated CAR T cells will also be addressed.

## Introduction

**Fig. 1:**
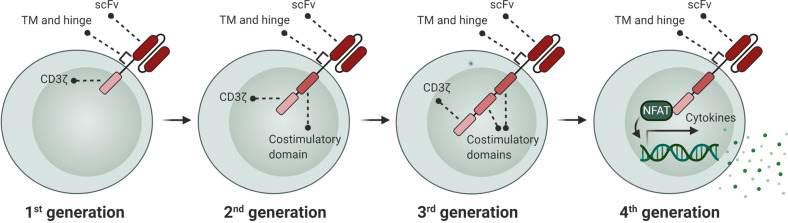
Development of CAR generations. CAR structure in CAR T cells compose of a single-chain variable fragment (scFv), a hinge and transmembrane (TM) region, costimulatory domain(s) (e.g., OX40, CD28, ICOS, 4-1BB), and a CD3ζ signaling domain. The 1st generation CARs contained only CD3ζ as intracellular domain, while the 2nd or 3rd generations have one or two costimulatory domains linked to CD3ζ, respectively. The 4th generation CARs are known as “TRUCK” CARs. These CARs are structurally similar to the 2nd generation CARs, but with an inducible cytokine expression (e.g., IL-12) through NFAT-responsive promoter.

In a recent decade, genetically modified immune cells, particularly chimeric antigen receptor (CAR) T cells, have raised enormous interest in clinical trials [1]. Several generations of CARs have now been developed that are different in the number of intracellular domains or CAR activation mode (Fig. 1). Despite the dramatic clinical benefit of CAR T cell therapy in a broad spectrum of cancer types, a large fraction of patients that achieves remission with CAR T cell therapy displays disease relapse within a few years [2, 3]. Several important explanations of treatment failure in CAR T cell therapies exist, such as tumor antigen escape and inefficient CAR T cell trafficking into the tumor site. However, it is widely thought that limited CAR T cell expansion and persistence in the hostile tumor microenvironment (TME) represent additional key impediments to efficacious CAR T cell responses and durable clinical remission following CAR T cell therapy [4]. The observed reduction in proliferative capacities and persistence of CAR T cells is associated with a generalized dysfunctional phenotype that is hallmarked by impaired proliferative and cytotoxic abilities. Importantly, the root cause for development of this dysfunctional state in CAR T cells is the activation of pathways that promote excessive CAR T cell differentiation, exhaustion, and senescence. Indeed, less-differentiated and less-exhausted CAR T cells have been reported to lead to a better outcome [5]. Central causes for in vivo induction of exhaustion and senescence are persistent stimulation of CAR T cells by high levels of tumor antigens in the face of chronic exposure to a suppressive TME [6]. At the same time, CAR T cell differentiation and exhaustion may be further accelerated by CAR antigen-independent tonic signaling [7].

Given the fact that the efficacy of CAR T cells depends on their capacity to infiltrate the tumor site and directly interact with tumor antigens, in vivo induction of exhaustion and senescence pathways is an unavoidable event in CAR T cell therapies. Indeed, once T cells are activated by the persistent antigen presentation, they subsequently become dysfunctional due to the elevated and sustained expression of inhibitory receptors [8]. Therefore, it is more attractive to focus on approaches to prevent intrinsic dysfunctional pathways in CAR T cells (e.g., inhibitory receptors signaling) and generate “exhaustion-resistant” cells, rather than aiming to modulate their exposure to tumor antigens in the TME. Through this strategy, the “exhaustion-resistant” CAR T cells might maintain their effector functions even during a prolonged exposure to their cognate antigen. In concordance with this, modified CAR T cells with disrupted pathways inducing exhaustion or senescence have shown a significantly higher persistence and antitumor activity, providing a promising outlook for reversal or delay of CAR T cell exhaustion and senescence as a way to harness the full potential of this highly effective treatment modality [9–11].

## Concept of exhaustion and senescence

Although exhausted T cells display some phenotypic markers that are typically associated with effector and memory states [12], they show phenotypically and functionally different properties from both effector and memory subsets [13]. Since exhaustion and senescence share several overlapping characteristics such as defective effector functions, impaired proliferation, and cell cycle arrest, they might be used interchangeably. However, there are certain characteristics that can be used to distinguish these states from each other, including cytokine secretion signatures, and expression of cell surface receptors and transcription factors [14].

**Fig. 2:**
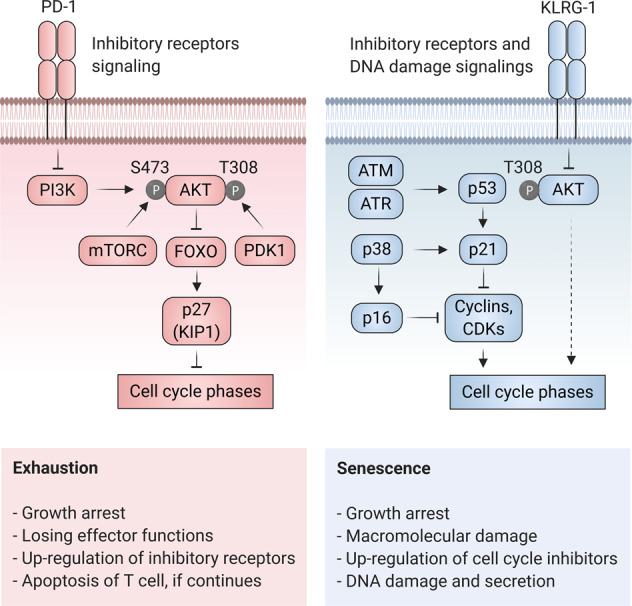
A scheme for induction of exhaustion and senescence. The principal feature of exhaustion (left) is upregulation of inhibitory receptors such as PD-1. Engagement of PD-1 to its ligand (PD-L1) results in the inhibition of intracellular signaling pathways involved in the regulation of cell proliferation such as PI3K/AKT pathway. Inactivation of AKT (due to the lack of site-specific phosphorylation mediated by PI3K, mTORC (on Serine473), and PDK1 (on Threonine308)) in turn lifts the block on FOXO transcription factors and thereby activates p27, a repressor of cell cycle (G1-S transition). Senescence (right) can be induced by either AKT inhibitory receptors (e.g., KLRG-1) or DNA damage response mediators including ATM and ATR. KLRG-1 prevents AKT phosphorylation (on Serine473) removing its block on p27, and thereby results in cell cycle arrest. The DNA damage response components activate p53, p21, p38, and p16 which inhibit cell cycle progression by blocking the function of cyclins and cyclin-dependent kinases (CDKs). The figure adapted from [16].

Recently, Wherry and Kurachi proposed a four-cell-stage model for T cell exhaustion that is initiated from TCF1^+^ exhausted T progenitors (Tex^prog1^, Tex^prog2^), and followed by “intermediate” (Tex^int^) and “terminally” (Tex^term^) exhausted subsets. They found that these cell subset transitions are regulated by the transcription factors TCF1, T-bet, and TOX in a hierarchical developmental pathway [15]. Exhausted T cells become dysfunctional via a progressive loss of functionality that is mainly mediated by upregulation of multiple inhibitory receptors, including for example PD-1, CTLA-4, TIM-3, which is typically observed in chronic infections and cancers [8, 16] (Fig. 2). However, expression of these inhibitory receptors is not an absolute indicator of T cell exhaustion, as they can also be induced on tumor-specific T cells upon activation when encountering their cognate tumor antigen [17]. Other major features of T cell exhaustion include modifications in T cell receptor (TCR) signaling, cytokine profile, pathways regulating migration, chemokine expression, and metabolic properties [18].

The molecular mechanisms governing induction of cellular senescence are still under investigation. Thus far, several types of senescence have been identified including, (i) replicative senescence or telomere-dependent senescence; (ii) DNA damage-induced senescence; (iii) oncogene-induced senescence; (iv) oxidative stress-induced senescence; (v) chemotherapy-induced senescence; (vi) mitochondrial dysfunction-associated senescence; (vii) epigenetically induced senescence; and (viii) paracrine senescence [19]. Replicative senescence is the most widely characterized senescence type, in which the cells acquire a poor proliferation potential after multiple divisions, and ultimately undergo total growth arrest as a consequence of telomere shortening [20]. In human T cells, the loss of the costimulatory molecules CD27 and CD28, and the high expression of coinhibitory receptors such as KLRG-1, CD57, and TIM-3 are prominent events in the replicative senescence [21]. Of particular interest, KLRG-1^+^ or CD57^+^ T cells are proliferation incompetent cells with decreased effector cytokine production that are susceptible to apoptosis [22, 23]. Cell cycle arrest through p21^WAF1/Cip1^/p16^INK4A^ pathway is a common feature of senescent cells which essentially acts as an alarm in response to damaging stimuli (e.g., DNA damage) or aberrant proliferation [24] (Fig. 2). It was previously thought that, in contrast to a quiescence state, a senescence-associated growth arrest is an irreversible event, but nowadays it is well established that early senescence and exhaustion stages are reversible [16]. Given this finding, blockade of senescence inducing pathways in CAR T cells may reinstate an enhanced proliferative capacity in a similar manner to blockade or reversal of exhaustion. However, blockade of key mediators of senescence (e.g., p38) in CAR T cells may still cause these cells to have a reduced ability to secrete effector cytokines IFNγ and TNF [25].

Collectively, both exhaustion and senescence can be regulated independently, and could be delayed or reversed in CAR T cells by targeting the specific pathways that govern their induction. In addition, drivers or repressors of exhaustion and senescence might affect various cellular pathways including, immune checkpoint, gene expression, signaling pathways, cell cycle progression, telomere maintenance, and oxidative stress pathways (Table 1). However, exhaustion blockade is assumed to be safer, since senescence acts as a tumor suppressor and efficiently safeguards the cells against damaging stimuli (e.g., DNA damage). Consequently, interventions to alleviate senescence might lead to the development of malignancy [16].

**Table 1:** Genes involved in the regulation of exhaustion and senescence pathways in T cells.

Gene name	Pathway	Mechanism of action	Study in CAR T
PD-1	Exhaustion	Immune checkpoint	Yes [9, 28–31, 35, 114–116]
CTLA-4	Exhaustion	Immune checkpoint	Yes [33, 34]
TIM-3	Exhaustion/senescence	Immune checkpoint	Yes [37]
LAG-3	Exhaustion/senescence	Immune checkpoint	No
CD160	Exhaustion	Immune checkpoint	No
VISTA	Exhaustion	Immune checkpoint	No
BTLA	Exhaustion	Immune checkpoint	No
KLRG-1	Senescence	Immune checkpoint	No
CD57	Senescence	Immune checkpoint	No
TIGIT	Exhaustion/senescence	Immune checkpoint	Yes [45]
2B4	Exhaustion	Immune checkpoint	No
CD39	Exhaustion	Immune checkpoint	No
CD73	Exhaustion	Immune checkpoint	No
c-JUN	Exhaustion	Gene expression regulation	Yes [11]
c-FOS	Exhaustion	Gene expression regulation	Yes [11]
JunB	Exhaustion	Gene expression regulation	Yes [11]
IRF4	Exhaustion	Gene expression regulation	No
BATF	Exhaustion	Gene expression regulation	Yes [11]
BATF3	Exhaustion	Gene expression regulation	Yes [11]
NFAT	Exhaustion	Gene expression regulation	No
Eomes	Exhaustion	Gene expression regulation	Yes [51]
T-bet	Exhaustion	Gene expression regulation	Yes [50]
TOX	Exhaustion	Gene expression regulation	Yes [55]
NR4A	Exhaustion	Gene expression regulation	Yes [10]
BLIMP1	Exhaustion	Gene expression regulation	No
TCF1	Exhaustion	Gene expression regulation	Yes [104]
**DNMT3A**	Exhaustion	Gene expression regulation	No
PI3K	Exhaustion/senescence	Signaling mediator	Yes [58, 61]
AKT	Exhaustion/senescence	Signaling mediator	Yes [57]
mTOR	Exhaustion/senescence	Signaling mediator, cell cycle regulation	Yes [5]
FOXO	Exhaustion/senescence	Signaling mediator, cell cycle regulation	Yes [50]
PTPN2	Exhaustion	Signaling mediator	Yes [68]
PP2A	Exhaustion	Signaling mediator	Yes [69]
LCK	Exhaustion	Signaling mediator	Yes [70]
SHP1/SHP2	Exhaustion	Signaling mediator	Yes [70]
A2AR	Exhaustion	Signaling mediator	Yes [76, 78]
PKA	Exhaustion	Signaling mediator	Yes [79]
TGFBR1/TGFBR2	Exhaustion	Signaling mediator	Yes [82–84]
PDK1	Exhaustion	Signaling mediator	No
PKC	Exhaustion	Signaling mediator	No
p38	Senescence	Signaling mediator	No
PIR-B	Exhaustion	Signaling mediator	No
p21^CIP1^	Senescence	Cell cycle regulation	No
p16^INK4A^	Senescence	Cell cycle regulation	No
p53	Senescence	Cell cycle regulation	No
RB	Senescence	Cell cycle regulation	No
hTERT	Senescence	Telomeres stabilization	Yes [87]
CAT	Exhaustion/senescence	Oxidative stress response	Yes [90]

## Targeting intrinsic regulators of exhaustion and senescence

### PD-1

PD-1 is the most common inhibitory receptor expressed on the surface of exhausted CD8 T cells, and its disruption is associated with reversal of T cell exhaustion as well as increased functionality against tumor cells [26, 27]. Previous data indicated that co-administration of PD-1 blocking antibody could enhance the therapeutic efficacy of CAR T cells [28]. However, this strategy might cause subsequent systemic toxicities. In a safer approach, Brentjens and colleagues coexpressed a PD-1-blocking single-chain variable fragment (scFv) in CAR T cells to boost their function and persistence in the TME. The blocking scFv markedly reinvigorated CAR T cells and restricted its effects mainly to the tumor site, thereby avoiding potential systemic toxicities [9]. In another proof-of-principle study, cytolytic function and cytokine secretion could be restored in exhausted CAR T cells by several approaches disrupting PD-1 interaction with PD-1 ligand (PD-L1), including a PD-1 blocking antibody, PD-1 silencing by RNA interference and co-transduction of a PD-1 dominant negative receptor [29]. The feasibility of cotransducing CAR T cells with a PD-1 decoy receptor has been also been proven by other researchers. For example, Huang and colleagues generated a modified B7-H3 directed CAR T cell coexpressing a PD-1 decoy receptor consisting of the extracellular PD-1 domain fused to the intracellular stimulatory domain of either CD28 or IL-7 receptor. This decoy CAR was able to convert PD-1 inhibitory signal to costimulatory signals [30]. The advent of CRISPR/Cas9 technology paved the way for additional ways to modify CAR T cells in the recent years. For instance, CRISPR/Cas9 technology was used by Lim et al. to generate PD-1-deficient anti-CD19 CAR T cells that were highly capable of eradicating tumor cells [31]. However, deletion of PD-1-encoding gene (*Pdcd1*) may also produce undesirable effects, since Wherry and colleagues reported that it unexpectedly promoted exhaustion and impaired T cell survival and function [32].

### CTLA-4

Like PD-1, inhibition of the PI3K/AKT pathway is the underlying molecular mechanism by which CTLA-4 induces T cell exhaustion [8]. A similar scenario can therefore be considered to reverse the dysfunctional state of CAR T cells induced by CTLA-4 pathway. Simultaneous blockade of CTLA-4, PD-1, and TIM-3 in CAR T cells by so-called blocking minibodies enhanced their effector functions. Among the different combinations of minibody-secreting CAR T cells, however, only the anti-CTLA-4 minibody-secreting CAR T cells showed prolonged function, signifying the unique characteristics of this immune checkpoint mediator [33]. Using a similar concept, checkpoint-resistant CAR T cells with genetic ablation for TCR, HLA class I, PD-1, and CTLA-4 were generated by a one-shot CRISPR system. However, the disruption efficiency minimized with increased numbers of target genes, which might be due to competition between multiple guide RNAs (gRNAs) for Cas9. In addition, the in vivo efficacy and lifespan of these PD-1- and CTLA-4-deficient CAR T cells need to be further examined [34].

### TIM-3 and LAG-3

Blockade of PD-1 in combination with other immune checkpoint receptors, including TIM-3 and LAG-3, has strong synergistic effects, and boosts effector functions of CAR T cells [35, 36]. The high efficacy of this combinatorial approach suggests that TIM-3 and LAG-3 pathways have non-redundant effects that synergize with PD-1 signaling to dampen antitumor responses in dysfunctional CAR T cells. As that the precise mechanisms by which TIM-3 and LAG-3 induce T cell exhaustion and senescence are still not fully understood, only a few studies have explored selective blockade of these inhibitory receptors in CAR T cells. In one effort, antibody-based blocking of Galectin-9, a putative TIM-3 ligand, reduced exhaustion of CAR T cells and significantly increased their cytotoxicity against previously resistant tumor cells [37]. Of note, FGL1 has been identified as a major ligand for LAG-3, and possibly will be considered as a candidate target for LAG-3 signaling blockade in the future studies [38]. Additionally, it has been speculated that intracellular trafficking and cell surface expression of LAG-3 is dependent on PKC signaling, revealing a role of PKC in mediating T cell exhaustion [39]. This notion is in contrast with the recent finding that exhaustion is reversed in T cells by PKC-inducing small molecules [40]. An explanation for this discrepancy is that the function of PKC-θ can be disrupted by PD-1/SHP-2-dependent CD28 inactivation, as CD28 is necessary for PKC-θ-mediated downstream signaling [41, 42]. Therefore, augmentation of PKC functioning might be useful for bypassing the suppressive PD-1 signaling. However, the distinct role of PKC, especially regarding different PKC isoforms, in T cell exhaustion seems to remain controversial.

### TIGIT

TIGIT is a recently identified immune checkpoint that is transiently overexpressed in activated dysfunctional CD8 T cells, regulatory T cells, and natural killer (NK) cells that mediates signaling for exhaustion and senescence in host cells upon binding to its ligands (CD155 and CD112) on the surface of antigen-presenting cells (APCs) [36, 43, 44]. Similar to TIM-3 and LAG-3, the knowledge about the intracellular signaling cascade activated by TIGIT during induction of exhaustion and senescence is largely incomplete. Since TIGIT competes with its costimulatory counterpart, CD226 (DNAM-1), for binding to the same ligands, engagement of TIGIT with its ligands diminishes costimulation signaling in the host cells. In line with this, concomitant expression of a TIGIT-based chimeric costimulatory switch receptor composing of the extracellular domain of TIGIT and the signaling domain of CD28, called TIGIT-28, endowed a superior antitumor function to the anti-CD19 CAR T cells, while rescuing the hypofunctional T cells [45].

### c-JUN/c-FOS axis

Dysregulation of AP1 transcription factor-binding motifs is a predominant epigenetic alteration in exhausted T cells, which leads to increased expression of exhaustion-related transcription factors from bZIP/IRF family (BATF3, IRF4) and/or AP1 family (BATF, JunB). These events subsequently elicit inhibitory signaling molecules in T cells, such as PD-1 [11, 46]. In a recent work, Mackall and colleagues overexpressed c-JUN, an AP1 family transcription factor involved in T cell activation, in CAR T cells to reverse the dysfunctional state of exhaustion-prone CAR T cells. They showed that CAR T cells with forced overexpression of c-JUN became exhaustion-resistant and displayed enhanced functional capacities as well as a reduced expression of exhaustion markers PD-1 and CD39. Mechanistically, c-JUN prevented development of terminally exhausted CAR T cells by directly activating AP1 complex (c-JUN/c-FOS), and indirectly disrupting AP1i complex (exhaustion-associated complex) through displacing JunB, BATF, and BATF3 from chromatin [11]. Another possible interpretation is that c-JUN competes for chromatin binding with NR4A family members, which regulate T cell exhaustion, as c-JUN and NR4A1 share a substantial number of common chromatin-binding sites [47]. Intriguingly, although c-JUN overexpression increased expansion of CAR T cells, it had no marked effect on cytokine production. Moreover, future studies are warranted to fully understand why overexpression of c-FOS alone, unlike c-JUN, did not lead to the enhanced functioning of CAR T cells [11].

### Eomes and T-bet

The transcription factors Eomes and T-bet play a key role in differentiation and exhaustion of T cells. Eomes knockdown restores the functional defects of exhausted T cells, indicating its positive impact on the development of T cell exhaustion. Conversely, T-bet^hi^ T cells express an intermediate level of inhibitory receptors, and are linked to the high functional properties [48, 49]. However, new data suggest that T-bet, in a key interplay between TCF1 and TOX, has a role in the development and dynamics of T cell exhaustion [15]. In the absence of IL-12, CAR T cells secreting IL-18 switched to a T-bet^hi^ FOXO1^lo^ phenotype that prevented exhaustion and augmented antitumor immunity [50]. In a case study, PD-1 blockade increased CAR T cell efficacy and expansion, and decreased Eomes expression, implying a positive correlation between PD-1 and Eomes expression in terminally exhausted CAR T cells [51].

### TOX/NR4A axis

The sustained expression of TOX, an HMG-box transcription factor involved in the regulation of thymocyte selection [52], in exhausted T cells is associated with impaired antitumor activity, and its downregulation alleviates T cell exhaustion [15, 53]. This transcription factor is induced by calcineurin and NFAT, and is implicated in the early epigenetic events responsible for fate commitment of exhausted T cells [54]. In addition to TOX, calcineurin-regulated NFAT can regulate the expression of NR4A, which cooperates with TOX proteins in a positive feedback loop to induce exhaustion in PD-1^hi^ TIM-3^hi^ CAR T cells [55]. Therefore, disruption of TOX and NR4A function may inhibit the induction of exhaustion in CAR T cells. In line with this, TOX- and NR4A-deficient CAR T cells demonstrated increased cytokine production and decreased expression of inhibitory receptors [55]. Moreover, NR4A triple knockout CAR T cells lacking NR4A1, NR4A2, and NR4A3 with a PD-1^hi^ TIM-3^hi^ phenotype exhibited a superior effector phenotype. Of note, chromatin accessible regions in these modified CAR T cells were selectively enriched for binding motifs for NFκB and AP1, which are actively involved in regulation of T cell effector functions [10].

### PI3K/AKT/mTOR/FOXO axis

In human lymphocytes, the PI3K–AKT pathway plays a critical role in regulating T cell differentiation and activity. Although this signaling pathway is essential for TCR signaling and production of cytolytic molecules [14], constitutively active AKT induces terminal differentiation and formation of KLRG-1^+^ effector T cells [56]. The advantage of AKT targeting is that there are numerous available AKT inhibitors compatible with CAR T cell expansion ex vivo. For instance, expansion of CAR T cells in the continuous presence of an allosteric kinase inhibitor (AKT Inhibitor VIII; AKTi) could generate minimally differentiated CD62L-expressing memory cells with superior antitumor function, without compromising cell yield [57].

It has been postulated that PI3K-mediated CAR T cell exhaustion is occurred through its role in tonic CAR signaling during ex vivo expansion, and this is why PI3K inhibitors preserve less-differentiated state of CAR T cells with heightened in vivo persistence [58]. PI3Ks are composed of four validated isoforms including, PI3Kα, PI3Kβ, PI3Kγ, and PI3Kδ, but only inhibition of PI3Kδ has resulted in a delay in terminal differentiation of T cells [59]. Consistent with this, mutation-derived hyperactive PI3Kδ decreased the threshold of exhaustion and senescence in T cells through cumulative epigenetic aberrations in the promoter region of inhibitory receptors (e.g., demethylation of the PD-1 promotor), leading to an increase in the inhibitory signaling [60]. This inhibitory effect was reported to be diminished in CAR T cells when PI3Kδ was selectively blocked by Idelalisib (CAL-101). The blockade switched CAR T cells to an undifferentiated phenotype (CD62L^+^ CAR T) that expressed lower levels of exhaustion markers [61].

The transcription factor mTOR is a multifunctional regulator of T cell metabolism that plays various roles in the regulation of exhaustion and senescence pathways. On the one hand, activation of mTOR, when accompanied by cell cycle arrest and DNA damage, promotes cell senescence [62, 63]. On the other hand, mTOR activation leads to a decreased cell exhaustion via declined FOXO function [16], a critical rheostat downstream of mTOR that sustains PD-1 signaling to promote the exhaustion of PD-1^hi^ Eomes^hi^ T cells [64]. Moreover, FOXO differentially regulates T-bet and Eomes, two transcription factors implicated in type I effector and differentiated memory phenotypes, respectively [65]. These findings support the notion that regulation of FOXO by PD-1 is necessary for induction of T cell exhaustion [16], and importantly, the whole process is augmented by a positive feedback loop between PD-1 and FOXO [64]. However, FOXO-deficient T cells fail to retain their persistence, indicating the functionally diverse mechanisms of this transcription factor in the regulation of T cell exhaustion and longevity [64, 65]. Thus, the impact of mTOR/FOXO axis on the T cell exhaustion/senescence is likely determined under specific circumstances. Preferably, targeting of mTOR/FOXO axis in the CAR T cells is performed by cytokine exposure. Ex vivo expansion of CAR T cells in the presence of IL-15 (or IL-2 with mTOR inhibitors) resulted in a less-exhausted stem cell memory T (T_scm_) phenotype with higher anti-apoptotic properties, and enhanced the proliferative capacity of CAR T cells. The authors argued that depletion of mTORC1 activity was a hallmark of the IL15-mediated harnessing of CAR T cell dysfunction [5]. In another platform, a FOXO1^lo^ T-bet^hi^ phenotype was induced in CAR T cells via inducible IL-18 expression, and the modified CAR T cells had superior antitumor function and prolonged survival [50].

### PTPN2 and PP2A

It has been recently established that the phosphatases PTPN2 and PP2A act as a critical regulator of CD8 + T cell exhaustion by attenuating the type 1 interferon pathway and mediating CTLA-4 signaling, respectively [66, 67]. Recently, stronger activation and improved tumor infiltration was observed in PTPN2-deficient CAR T cells, which were less prone to exhaustion. The authors concluded that this “exhaustion-resistant” phenotype of CAR T cells was obtained by upregulation of LCK and cytokine-induced STAT-5 signaling [68]. However, suppressing CAR T cell exhaustion by PTPN2 ablation might not always have desirable output, as for example deletion of the PTPN2-encoding gene (*Ptpn2*) in CD8 T cells enhanced the ratio of terminally exhausted T cell populations (TIM-3^+^ T cells) to progenitor exhausted T cells (SLAMF6^+^ T cells) by increasing the number of TIM-3^+^ cells without depleting the pool of SLAMF6^+^ populations [66]. To disrupt PP2A function, Zhuang and colleagues used the small molecule LB-100, and found that inhibition of this phosphatase increased the therapeutic efficacy of CAR T cells [69].

### LCK and SHP1/SHP2

SHP1 counteracts the effect of LCK and dephosphorylates key components of TCRs and CARs including CD3ζ, specifically in BBζ CARs [70]. Although dispensable, SHP2 is also considered as a molecule involved in development of T cell exhaustion [71]. These phosphatases are recruited to the cytoplasmic portion of PD-1 and other inhibitory receptors, and negatively regulate IL-2 production upon PD-1 engagement [72]. Some preliminary data are available that report the feasibility of SHP1 ablation in CAR T cells [73, 74]. In a recent report, Dotti et al. engineered LCK and SHP1 to be recruited within the synapse of T cells expressing BBζ and 28ζ CARs, respectively. The results indicated that overexpression of LCK in CAR T cells encoding 4-1BB could boost the expansion capacity and antitumor functions. Remarkably, LCK-mediated CAR T cell activation did not show any increase in expression of inhibitory receptors. In contrary, recruitment of SHP1 to the synapse of CD28-encoding CAR T cells by heterodimerization of the small molecule AP21967 did not augment antitumor effects, but decreased the severity of cytokine release syndrome (CRS) [70]. These findings highlight the potential of LCK and SHP1 for generating CAR T cells with predictable activity and toxicity profiles, respectively.

### A2AR/PKA axis

In the hypoxic conditions, the HIF-1α pathway is amplified in tumor cells, which leads to upregulation of CD73 and CD39, two ectonucleotidases involved in adenosine signaling on the surface of both tumor and T cells [75]. Adenosine is known as an immunosuppressive molecule that exerts an inhibitory signal within T cells following its A2AR-mediated delivery into cytoplasm and subsequent conversion to the cyclic AMP (cAMP). In the TME, CAR T cells upregulate A2AR that predominantly suppresses endogenous antitumor responses via facilitating the adenosine-to-cAMP conversion [76]. Inhibition of A2AR results in greater expansion of tumor-specific CD8 T cells [77], which suggests that this receptor may represent a highly translational target for blocking T cell exhaustion. As shown independently by two groups, pharmacological targeting of A2AR or expression of an anti-A2AR short hairpin RNA (shRNA) in CAR T cells restored their proliferation and cytokine production deficits caused by the adenosine signaling [76, 78]. In association with Ezrin, PKA sustains the inhibitory effect of adenosine signaling by blocking TCR signaling in CAR T cells. Previous data showed that expression of a peptide-based inhibitor of PKA and blocking of Ezrin association blunted the negative effect of adenosine/PKA/Ezrin axis in CAR T cells [79].

### TGFBR1/TGFBR2

TGF-β secretion in TME is particularly evident in resistant tumors, and is associated with poor antitumor immunity and clinical outcome [80]. Of particular interest here, activation of TGF-β signaling in tumor-reactive T cell acts as a potent inducer of exhaustion pathways via phosphorylation of SMAD2 and SMAD3 ahead of immune checkpoints. This reflects a hierarchy of inhibitory events during T cell exhaustion that is initiated by TGF-β signaling cascade and that subsequently progresses due to PD-1/PD-L1 interaction [81, 82]. To address this, various immunotherapies based on blocking TGF-β (signaling) are in clinical development, which holds promise for revitalizing T cell antitumor activity in resistant tumors [81]. In CAR T cell therapy, these approaches have focused on targeting TGF-β receptors. For example, Wang et al. knocked out the endogenous TGFBR2 in CAR T cells with CRISPR/Cas9 technology to prevent exhaustion by blocking TGF-β-induced phosphorylation of SMAD proteins. Impressively, knocking out TGFBR2 bestowed CAR T cells with superior proliferative and tumoricidal capabilities, both in vitro and in vivo [82]. In another attempt, a dominant-negative TGFBR2 was coexpressed in PSMA-specific CAR T cells. The engineered CAR T cells were resistant to exhaustion, while they displayed enhanced abilities to proliferate and secrete cytokines. However, the enhanced proliferation of CAR T cells was associated with lymphoproliferative syndrome, emphasizing the safety considerations to be addressed before employing this approach [83]. A successful TGF-β signal conversion platform has also been reported in recent years. In this approach, T cells were transduced with chimeric variants of TGFBR1 and TGFBR2 containing the TGF-β-binding domain of each receptor and the intracellular signaling domains of a T cell stimulating interleukin receptor. As anticipated, TGF-β exposure induced STAT signaling (instead of SMAD2/3 signaling), and promoted effector function and persistence of CAR T cells [84].

### hTERT

Endogenous hTERT, which encodes the catalytic component of the telomerase complex, serves as a major determinant of human T cell longevity. Expression of this gene is decreased in the memory T cells upon antigen encounter [85]. It has been indicated that hTERT downregulation directly imposes replicative senescence in terminally differentiated T cells [20]. Having observed these findings, ectopic hTERT expression in T cells restored gradual loss of telomeric DNA, and extended the lifespan of T cells without loss of functionality [86]. As yet, limited data has been obtained in the CAR T cells. In an attempt, Bai et al. could successfully increase the proliferative capacity of CD19-directed CAR T cells using a transiently coexpression of hTERT. The hTERT upregulation improved antitumor effects of CAR T cells, and delayed initiation of replicative senescence [87].

### CAT

Although low concentrations of reactive oxygen species (ROS), most notably H_2_O_2_, act as signal molecules and trigger intracellular signaling as well as defense pathways against pathogens, increased levels induce cell senescence and exhaustion pathways [88]. Effector T cells encountering the hostile TME or viral infections are skewed toward state with reduced effector functioning, reduced glucose uptake and glycolysis, upregulated inhibitory receptors, and increased ROS levels [89]. This gives rise to the rationale that blockade of high-level ROS can reinvigorate CAR T cells in vivo. Indeed, CAR T cells coexpressing CAT enzyme, an antioxidant enzyme that specifically scavenges H_2_O_2_, were largely protected against intrinsic and extrinsic oxidative stress, and demonstrated higher persistence and efficacy [90]. Even though increased ROS levels in the TME are generally unfavorable for CAR T cell function, it has been shown that pre-treatment of cancer cells with a ROS accelerator renders them more susceptible to CAR T cell-mediated killing. These ROS accelerators did not affect CAR T cell function, opening a window of opportunity for combinational treatment using armored CAR T cells and ROS accelerators [91].

**Fig. 3:**
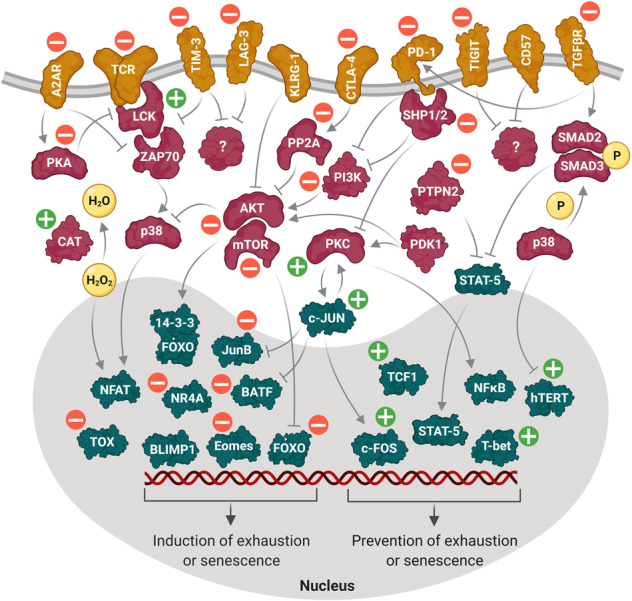
Intracellular network between exhaustion or senescence regulators. Competition between exhaustion or senescence regulators and inhibitors determines the fate of CAR T cell in the TME. Inhibitory receptors transfer exhaustion/senescence signal to the inhibitory transcription factors (i.e., FOXO, Eomes, BATF, BLIMP1, TOX, NR4A, IRF4, NFAT, etc.) via downstream signaling pathways, whereas exhaustion/senescence delaying transcription factors (i.e., c-FOS, TCF1, T-bet, NFκB, etc.) counteract these inhibitory signals. The green plus and red minus signs refer to the enhanced/upregulated and blocked/downregulated genes in CAR T cells, respectively.

Overall, prevention of CAR T cell dysfunction through blockade of major drivers of exhaustion and senescence, or by augmentation of repressors of exhaustion and senescence has led to some appealing results that could potentially contribute to improved CAR T cell-based immunotherapies. The considerable number of genes targeted for this purpose in CAR T cells, either by genetic modification or pharmacological interventions, are depicted in Fig. 3.

## Costimulation induction and cytokine exposure

**Fig. 4:**
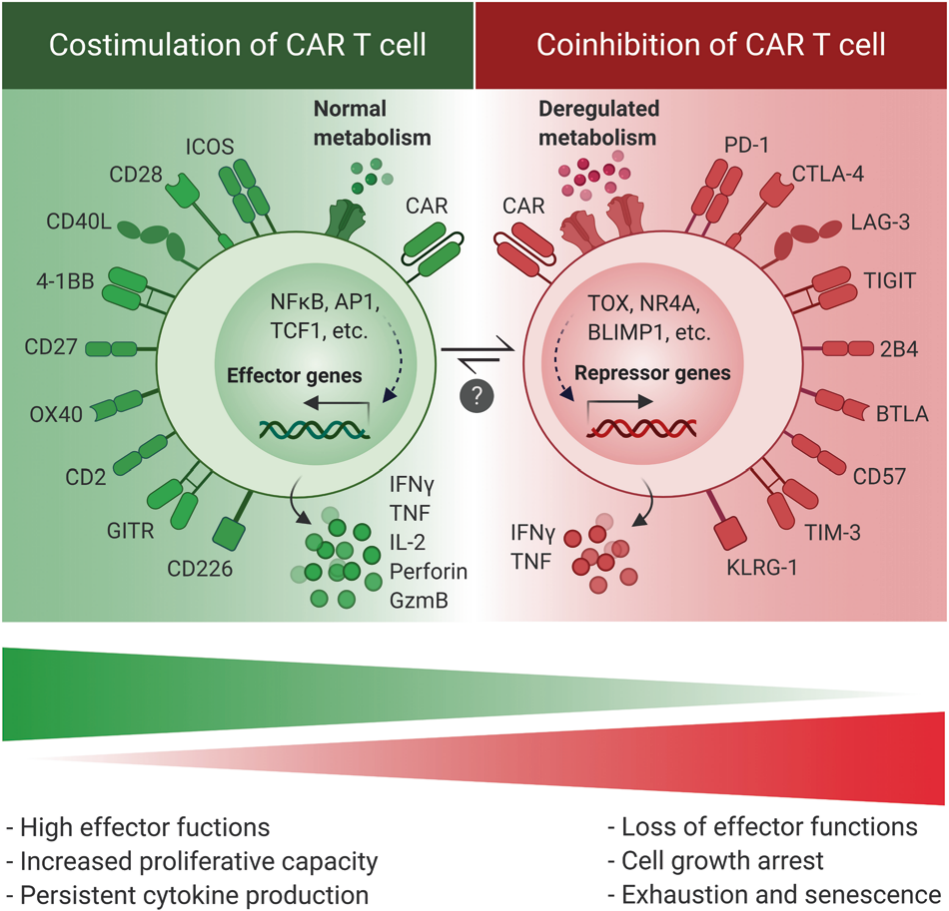
Costimulation and coinhibition of CAR T cell. Upregulation of costimulatory and coinhibitory receptors on the surface of CAR T cells is associated with effector polyfunctional and exhausted hypofunctional phenotypes, respectively. The final functional outcome depends on the number and diversity of relevant receptors. Although differentiation of functional CAR T cells to the exhausted/senescent phenotype is a common phenomenon in the tumor site, the possibility of reinvigorating exhausted/senescent CAR T cells is still a question.

After priming of T cells by antigens, costimulatory factors, and inflammation mediators, they can subsequently differentiate into two new subsets: (i) highly polyfunctional memory T cells, enabling them to become vigorously cytolytic with high proliferative potential; (ii) dysfunctional T cells with almost no effector functions, which show a progressive increase in the number, diversity, and “intensity” of inhibitory receptors [13] (Fig. 4). Therefore, differentiation of functional CAR T cells into exhausted or senescent stage is a predictable phenomenon in the tumors with suppressive barriers and high antigen load. But a question emerges: whether the costimulation induction and cytokine exposure can prevent or delay the onset of exhaustion or senescence of CAR T cells? To address this, several studies have demonstrated an enhanced persistence and efficacy in the CAR T cells when exposed to the costimulatory molecules or cytokines [7, 92–94].

CD28 and 4-1BB intracellular domains are the most widely used domains for generation of CARs, which are associated with prompt effector response and ﻿ long-term function, respectively [95]. New costimulatory factors have also shown to have a role in the persistence of CAR T cells. As suggested by June et al., incorporation of ICOS (also called CD278) and 4-1BB intracellular domains into CARs remarkably augmented the effector function and persistence of CAR T cells. Interestingly, the strong costimulatory effect was observed only when ICOS domain was linked to its transmembrane peptide, and thereby positioned proximal to the cell membrane [92]. This demonstrates that proper selection and configuration of costimulatory domains is an essential consideration. In functional terms, ICOS-bearing CARs were initially identified to preserve the signature characteristics of CD4 T_H_17 cells. This costimulatory domain could induce antitumor responses (by upregulating IFNγ) and mitigate exhaustion (by upregulating T-bet) in T_H_17 cells carrying a CAR [96]. This finding led to the extension of ICOS-based CARs to the CD8 T cells [92].

Previous studies suggest that antigen-independent tonic signaling by CARs, perhaps due to the physical interactions between CARs or scFv dimerization, limits CAR T cells potency by induction of exhaustion pathways. This antigen-independent exhaustion can be tolerated by CAR T cells through tonic 4-1BB signaling. [97]. However, other parts of the CAR construct design as well as the transduction method used to generate CAR T cells can also contribute to the extent of tonic signaling, and the effect of CD28/4-1BB domains should be evaluated for each individual CAR [7, 97]. Recruitment of TRAF proteins and induction of noncanonical NFκB signaling are critical events in 4-1BB costimulation, and contribute to mitigated exhaustion [98, 99]. Importantly, endodomains of CD28 may promote antigen-independent exhaustion, since replacing of this costimulatory molecule with 4-1BB reversed exhaustion in CAR T cells [7]. Recently, Posey et al. reported that a single asparagine (Asn) amino acid in the intracellular domain of CD28 is responsible for CD28-mediated exhaustion in CAR T cells. Substituting this Asn to phenylalanine hindered cell exhaustion and led to a durable antitumor response [100]. Based on these findings, although induction of CD28 signaling will apparently restore the immunocompetence of senescent CAR T cells [101], it might drive the cells into states of exhaustion and dysfunction. Therefore, the mutant-type CD28 (with substituted Asn in the YMNM motif) costimulation in CAR T cells may result in more benefits [100].

Cytokines are able to provide further stimulatory signals for T cell activation, and therefore have been effectively used to exert a spectrum of pleiotropic effects on CAR T cell proliferation and function [102]. Apparently, the rationale underlying this approach’s efficiency is that cytokines are able not only to convert the inherent features of hypofunctional CAR T cells to the effector signature, but also to reduce the number of suppressor cells in TME, resulting in an augmented immunity [50, 93, 94, 101, 103]. Apart from IL-2, the most widely used cytokine to culture CAR T cells for ACT, a number of other cytokines such as IL-7, IL-15, IL-18, and IL-21 have shown promising outputs [93, 94, 102, 103]. In a clinical setting, Dotti et al. demonstrated that IL-7 and IL-15 preserved more CAR T cells with T_scm_ phenotype. High frequency of these less-differentiated CAR T cells was correlated with overall in vivo expansion and persistence in lymphoma patients [93]. Although controversial, IL-15 has the advantage of having synergistic effect with different cytokines [5, 93, 104]. Simultaneous coexpression of IL-15 and IL-21 in CAR T cells enriched for less-differentiated T cells by sustaining TCF1 expression, a transcription factor associated with higher percentage of stem cell memory and central memory populations [104]. In addition, IL-18-secreting CAR T cells have exhibited a boosted proliferation and antitumor activity, describing the IL-18 influence on the CAR T cell function and persistence [103, 105].

## Change in the milieu of TME

External suppressive signals (i.e., TGF-β, IL-10, PGE2, soluble FAS, adenosine, ROS) from stromal and suppressive immune cells such as cancer associated fibroblasts (CAFs), myeloid-derived suppressor cells (MDSCs), tumor associated macrophages (TAMs), tumor associated neutrophils, mast cells, and regulatory T cells (T_reg_) contribute to the initiation of exhaustion of CAR T cells [106]. However, also tumor cells produce many of these suppressive mediators in the TME (i.e., PD-L1, TGF-β, IL-10, PGE2) to counteract effective immune responses. Activation of negative signals in tumor cells synergizes with the function of suppressive cells in the TME and can additionally induce a plethora of immunosuppressive pathways that may lead to the CAR T cell dysfunction [107]. Several efforts have been made to interfere with these suppressive signals activated by either tumoral or non-tumoral cells to make the TME milieu more tolerable for CAR T cells. Long and colleagues modulated MDSCs by all-trans retinoic acid (ATRA) molecule to diminish the suppressive effect of these cells on CAR T cells. Co-administration of ATRA enhanced antitumor effect of CAR T cells and prolonged their survival in vivo [108]. Aiming to eliminate MDSCs from the TME, Parihar et al. described a creative strategy by means of NKG2D-based CAR NK cells. The authors found that pretreatment of xenograft model with the modified NK cells resisted CAR T cells to the TME suppression, and improved tumor infiltration and efficacy of CAR T cells through killing NKG2D ligand-expressing tumor cells and MDSCs. Of note, administration of CAR T cells alone could not prevent tumor growth due to the suppressive signals from tumor-infiltrated MDSCs [109]. Blockade of suppressor cells in the TME can also lead to the elimination of immune checkpoint signaling, as these cells are an important source of immune checkpoint ligands (i.e., PD-L1). In concordance with this, targeting PD-L1-positive MDSCs and T_regs_ in the TME prevented CAR T cell suppression by these cells’ immunosuppressive pathways, and augmented the efficacy of CAR T cell therapy [110]. Some studies have focused on the enhancement of CAR T cell resistance to the suppressor cells, rather than direct targeting of suppressor cells. As an instance, CAR T cells with constitutive or induced expression of IL-12 switched to the resistant cells with less susceptibility to T_regs_, MDSCs, and TAMs [111–113]. Notably, the IL-12-mediated stromal collapse was associated with the upregulation of FAS death receptors on MDSCs, macrophages, and dendritic cells (DCs) within the tumor stroma [112].

Targeting specific surface markers of cancer-associated stromal cells (CASCs) such as PD-L1 have the potential to reinforce CAR T cell therapies in tumor site by collapsing dense stromal components [114]. In an effort, co-administration of CAR T cells with oncolytic adenoviruses expressing PD-L1 blocking antibodies not only resulted in further antitumor activity, but also diminished the expression of PD-1 on the surface of CAR T cells [115]. In addition, tumor site-specific delivery of PD-L1 antibody by CAR T cells could impede the cell exhaustion and diminish tumor growth much better than conventional CAR T cells [116]. In an interesting approach, PD-L1 in the TME was targeted as an antigen via nanobody-based CAR T cells. The PD-L1-targeted CAR T cells successfully controlled tumor growth and alleviated the expression of exhaustion markers such as LAG-3, TIM-3, and PD-1 [114]. This approach has two advantages of activating local antitumor response in the TME and eliminating CASCs, simultaneously. As highlighted above, TGF-β is another critical suppressor of effector T cells that is produced by major cell compartments in the TME. Thus, antagonizing this ligand may benefit cancer immunotherapies [80]. Efforts including, CD28 or cytokine costimulation, expression of constitutively active AKT, expression of dominant negative TGF-β receptors, or design of TGF-β-based decoy CARs have been carried out to overcome TGF-β-mediated repression of CAR T cells [117]. In addition to PD-L1 and TGF-β, tumor cells and some immune cells in the TME can further suppress CAR T cells by expressing IDO, an intracellular enzyme that converts the amino acid tryptophan to the kynurenine. The immunosuppressive kynurenine subsequently increases T_regs_ population and biases the TME’s cells (i.e., DCs and macrophages) toward an immunosuppressive phenotype through activating the receptor AhR [118–120]. Downregulation of IDO by fludarabine and cyclophosphamide, two lymphodepleting drugs often used before CAR T cell infusion, significantly improved the efficacy of CAR T cell therapy in IDO-positive tumors [121]. The serine protease FAP, which is highly expressed by CASCs like CAFs, may also play an important role in alleviation of antitumor immunity. Direct targeting of this protease using CAR approach has shown to be safe and effective for depleting the FAP^hi^ stromal cells and enhancing CAR T cell function [122]. Lately, FAP-specific CAR T cells advanced into a phase I clinical trial for treatment of patients with malignant pleural mesothelioma (NCT01722149). TAMs, particularly M2 polarized macrophages, express several specific markers that can be modulated by CAR-based modalities. CSF1R is one of such TAM biomarkers that was recently targeted using CAR T and CAR NK platforms, and represented a promising potential for depletion of the suppressive TAMs [123]. Further TAM biomarkers (i.e., MARCO, FRβ) are also being interrogated as potential candidates for TAM targeting to establish optimal CAR T immunotherapies [124].

It should be noted that metabolic changes in the TME can enforce the exhausted phenotype of CAR T cells. Indeed, due to the limited vascular exchange and metabolism of tumor cells leading to a microenvironment with acidic, hypoxic and/or depleted nutrients (e.g., glucose, amino acids), the TME is metabolically hostile [125]. Metabolic dysfunction in the tumor site linked to early inhibitory signals can enhance the development of T cell exhaustion [126]. As it happens with cancer cells, T cell metabolism is totally dependent on the composition of the TME, which may enable the necessary activity levels of aerobic glycolysis, pentose phosphate and tricarboxylic acid pathways required for T cell proliferation and function. For instance, under low-glucose conditions T cells produce lower levels of effector molecules such as IFNγ, granzyme B, and IL-7 [127, 128]. Another important aspect is mitochondrial integrity and number, which is crucial for effector cells. Exhausted tumor-infiltrating T cells show signs of mitochondrial dysfunction that correlate with reduced activity of PGC1α and the antitumor activity could be rescued by through overexpression of PGC1α [129]. Furthermore, production of immunosuppressive metabolites also contributes to the effector dysfunction of T cells. As an example, adenosine is a key metabolic regulator implicated in the T cell exhaustion. This metabolite is generated by the ectonucleotidases CD39 and CD73 and has a critical role in mediating suppressive effects of stromal cells, tumor cells, and infiltrating immune cells [75]. Hence, pharmacologic or genetic metabolic reprogramming of either the TME cells or CAR T cells is beginning to accrue. To this end, potential targets have been identified such as IDO, ARG1, CD36, CD39, CD73, GLS, A2AR, AMPK, and PDHK1 [130].

## Safety considerations and concluding remarks

It should be pointed out that reversing or delaying exhaustion and senescence in CAR T cells is a two-edged sword. Regardless of impressive efficacy of reinvigorated CAR T cells, a risk assessment of unexpected toxicities has remained to be investigated [11]. “On-target, on-tumor” toxicity can be a direct consequence of reinvigorated CAR T cells, which is related to the induction of CRS and tumor lysis syndrome induced by abundant cytokine release and excessive tumor cell death in tumor site [131]. On the other hand, a catastrophic and rapid “on-target, off-tumor” toxicity might be observed after infusion of armored CAR T cells targeting a tumor-associated antigen (TAA), which is expressed in both tumor and normal cells. This is the most serious complication of armored CAR T cells that results from a direct attack to the TAAs as well as long-term circulation and persistence of armored CAR T cells [131, 132]. Although controversial, senescence is considered to be a crucial obstacle against cancer development, and blockade of senescence drivers in tumor site may therefore be pro-carcinogenic [133, 134]. Taken together, there is a concern regarding the side effects associated with exhaustion/senescence-resistant CAR T cells. To avoid these adverse effects in modified CAR T cells, further developments need to be considered [135]. For instance, some researchers recommend to insert an inducible “suicide gene” or “elimination gene” (e.g., HSV-TK, inducible caspase 9, truncated EGFR, TMPK) into the CAR T cell genome that renders CAR T cells susceptible to an exogenous molecule-mediated death in case of adverse effects [136].

In conclusion, the field of cancer immunotherapy has undergone a revolution, with the development of adoptive cell transfer (ACT) therapies and immune checkpoint inhibitors (ICIs) leading the way in improving the outcome of cancer therapy. Nevertheless, the ability of the immune system to fight tumors in the hostile TME remains a major challenge for both ICIs and ACT therapies, particularly when it comes to various types of solid tumors. Here we summarized how the efficacy of CAR T cell therapy is restricted by the activation of exhaustion and senescence pathways, and what efforts have been made to reinvigorate dysfunctional CAR T cells. As research on CAR T cell dysfunction, especially in relation to the senescence, is a relatively new field and experimental data is limited, this review largely focuses on CAR T cell exhaustion. Approaches to delay the exhaustion/senescence in CAR T cells include direct modulation of intrinsic pathways and/or harnessing exogenous signals. Costimulation induction and cytokine exposure as well as attenuation of TME’s suppressive milieu appears to be feasible in this context. The efficacy and scalability of much of what has been discussed here is not limited to the CAR T cells, and can also be developed to other types of T cells therapies such as tumor-infiltrating lymphocytes (TILs) or TCR-engineered T (TCR-T) cells. However, further investigations into the safety of these platforms have to be taken into consideration.
